# Highly Photosensitive Colloidal Quantum Well Based Nanocrystal Skins Assisted by Orientation Control

**DOI:** 10.1021/acs.nanolett.5c03234

**Published:** 2025-11-17

**Authors:** Furkan Isik, Taylan Bozkaya, Iklim Bozkaya, Savas Delikanli, Betul Canimkurbey, Hilmi Volkan Demir

**Affiliations:** † UNAM − Institute of Materials Science and Nanotechnology and The National Nanotechnology Research Center, Bilkent University, Ankara 06800, Turkiye; ‡ LUMINOUS! Center of Excellence for Semiconductor Lighting and Displays, School of Electrical and Electronic Engineering, School of Physical and Mathematical Sciences, School of Materials Science and Engineering, Nanyang Technological University, Singapore 639798, Singapore; § Department of Electrical and Electronics Engineering, Department of Physics, Bilkent University, Ankara 06800, Turkiye; ∥ Institute of Accelerator Technologies, Ankara University, Ankara 06830, Turkiye; ⊥ Turkish Accelerator and Radiation Laboratory (TARLA), Ankara 06830, Turkiye; # Department of Physics, Polatlı Faculty of Arts and Sciences, Ankara Hacı Bayram Veli University, Ankara 06900, Turkiye

**Keywords:** colloidal quantum wells, light-sensitive nanocrystal skins, photosensing, self-assembly, charge carrier transfer

## Abstract

Colloidal quantum wells (CQWs) exhibit superior optical characteristics, including giant oscillator strengths and high absorption cross sections, making them attractive for light-sensing applications. In this study, we fabricated light-sensitive nanocrystal skin (LS-NS) devices using a single-layer edge-up oriented CQW film as the active absorber and achieved a significant enhancement in the performance by leveraging the controlled orientation of the self-assembled CQWs. The LS-NS devices with edge-up oriented CQW film show eight times higher sensitivity and three times higher voltage build-up compared to LS-NS devices with spin-coated CQW films. Such enhancements are due to the trapping of the holes on the edges of the CQWs because of defect-ridden edges, which helps hopping of the holes localized within close proximity to the metal–CQW interface. The LS-NS device with edge-up CQW film exhibited a record-high photovoltage buildup of 600 mV, three times greater than the highest previously reported value for similar LS-NS architectures.

## Graphical abstract

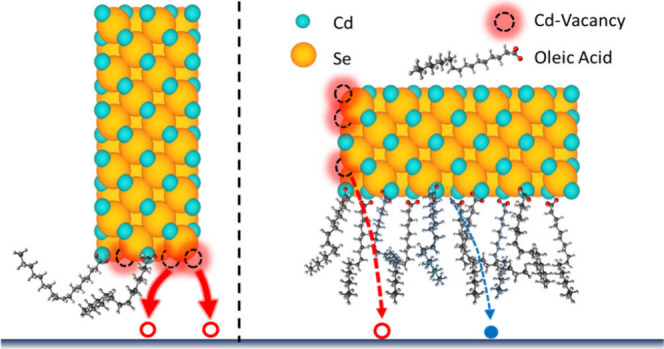

Colloidal semiconductor nanocrystals (CSNCs) are a promising class of materials for next generation optoelectronic applications, including light-emitting devices and photodetectors, owing to their widely tunable photophysical properties and low cost of production.^1^ Moreover, CSNCs can be implemented on any kind of substrate and matrix, allowing development of more sophisticated device structures via simple yet effective large-area thin-film techniques such as dip coating,^2^ blade coating,^3^ spray coating,^4^ and inkjet printing.^5^ Colloidal quantum wells (CQWs) are a quasi-2D subclass of CSNCs with superior photophysical properties owing to their 1D confinement such as giant oscillator strength, large absorption cross-section, and intrinsically suppressed Auger recombination, which are beneficial for light-absorbing applications.^6^ Thinner CQWs possess high exciton binding energy, which can be detrimental for photosensing applications as charge carrier separation is suppressed. However, this can be avoided by using thicker and type-II CQWs, which have much lower exciton binding energy,^7–10^ or appropriately choosing charge acceptors which can extract carriers on time scales far shorter than recombination.^11^ Moreover, their well-defined anisotropic structure with flat surfaces allows their self-assemblies with two distinct orientations (face-down and edge-up),^12–14^ which expose the different facets having different dimensions, surface structures, and ligand densities,^15–17^ making them an ideal platform for model studies for charge transfer phenomena.^18–20^

Light detectors, in general, work under external bias to extract photogenerated carriers, which in turn results as a current read-out.^21–23^ In CSNC light detectors, to achieve high sensitivity (high current read-out), the level of absorption should be high, which can be obtained by having thicker CSNC films as the active matrix. However, as the CSNC film gets thicker, we face charge hopping limitations due to short diffusion lengths and short carrier lifetimes of CSNCs, which results in high noise levels (dark current) and hence limited detection capability.^24,25^ Moreover, the external bias may adversely affect the device layers and CSNCs through electrochemical redox reactions.^26^ An alternative architecture for photodetectors is so-called light-sensitive nanocrystal skins (LS-NS). Their working principle relies on photogenerated voltage buildup without any requirement of external bias, and even a single layer of CSNC suffices to achieve high signal-to-noise ratios.^24,27–33^ However, the response of these LS-NS devices to a CW light source is limited to only a short voltage pulse, which might limit its practical usage. To overcome this limitation, a brief bias can be applied to neutralize and rapidly reset the device. The versatility of these power-efficient LS-NS devices has been demonstrated on transparent, large-area, flexible, and fragmentable substrates.^24,27,31^ The structure of a nanoskin is composed of a CSNC layer sandwiched between a dielectric and a metal layer. The circuit is completed with a Shunt resistor of 100 MΩ connecting the metal contact and dielectric layer. Even a very small amount of charge accumulation is enough for large photovoltage buildup on the order of 100 meV for these devices. In addition, even a single layer of CSNC is enough to generate high voltage, and noise is reduced owing to the elimination of complications by thick nanocrystal films.^24,27–33^ CQWs having facets with different dimensions and ligand densities provide an extraordinary platform for LS-NS devices.^28^ CQWs are prone to trapping of the holes on undercoordinated Se sites,^34,35^ which are especially abundant an the narrow facets.^15^ Such trapped carriers, which are detrimental for light-emitting applications, such as lasers and light-emitting diodes, may provide great opportunities for light-harvesting applications by carefully designing the device architectures.

In this study, we fabricated light-sensitive nanoskins having edge-up oriented single-layer self-assembled CQWs as an active layer and compared their performance with the light-sensitive nanoskins having a spin-coated CQW layer. We achieved record-high voltage build up values reaching 600 mV from the devices having an edge-up oriented single layer of CQWs, which is three times higher than the best reported value obtained with advanced LS-NS device architectures.^30^ Meanwhile, the sensitivity of the device reaches to 600 V/W, which is the highest among LS-NS devices with similar structures^24,31,33^ and in a range similar to the ones with advanced structures, exploiting exciton funneling, tandem structure, or plasmonic enhancement.^27–30,32^ The LS-NS devices with edge-up oriented film significantly outperformed the one with spin-coated film with up to nearly 8-fold sensitivity enhancement. Here, we attributed this improvement to the difference in the distribution and density of ligands on various facets (large and small), which affected the charge separation and hole hopping efficiency between the CQWs and the metal contact. Here, the orientation control allowed us to place defect-rich narrow facets of CQWs, which mediate the hole trapping, close to the contact layer, which in turn facilitated the efficient hole transfer and the record-high voltage build up. These results present the merits of orientation control of CQWs, which were demonstrated in LS-NS devices for light-sensing applications to be implemented in large-area photosensitive windows and glass facades of smart buildings and smartphones.

We fabricated LS-NS devices by integrating edge-up oriented CdSe CQWs as the absorber, exploiting anisotropic carrier dissociation at exposed facets. The device stack (Figure 1a) comprises an ITO (bottom electrode)/50 nm Al_2_O_3_ insulator/CQW film/Al (top electrode), with ITO and Al connected externally through a Shunt resistor. As an initial step in this study, we synthesized 4.5-monolayer-thick CdSe CQWs, which are well-established species of CQWs in terms of their synthesis, physical, and chemical properties, and hence they are one of the most appropriate classes of CQWs for proof-of-concept demonstrations and model studies^36^ (for the synthesis protocol see the Supporting Information). To fabricate the device, we first deposited a conformal 50 nm Al_2_O_3_ blocking layer by atomic-layer deposition on an ITO-coated glass. We then transferred the self-assembled monolayer of CQWs onto Al_2_O_3_ via a simplified Langmuir–Blodgett self-assembly, yielding a closed-packed edge-up orientation. To isolate orientation effects, we deposited CQW films by spin-coating the same CQWs, resulting in randomly oriented platelets. Absorbance spectra were matched between both film types to ensure identical photon capture and exciton generation. Finally, a 100 nm Al layer was thermally evaporated to define the top contact. Comparative device tests therefore probe how CQW orientation alone influences charge separation and voltage build-up in LS-NS architectures.

**Figure 1:**
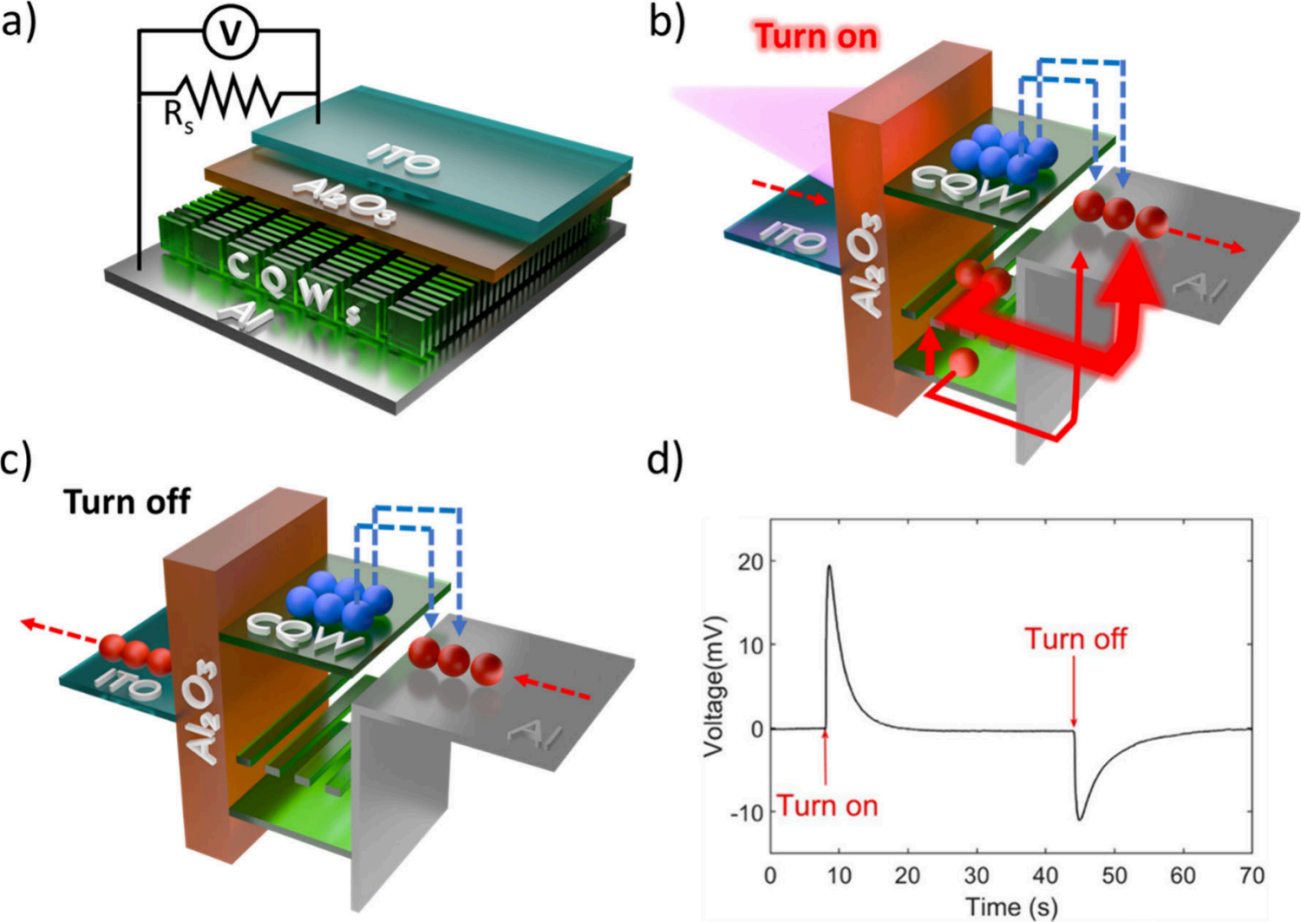
a) Illustrative scheme of the experimental setup, which also shows the layers of the vertical LS-NS device of CQWs. b) Simplified scheme of carrier transfer throughout the excitation phase of the photovoltage buildup principle. c) Simplified scheme of carrier transfer of the photovoltage buildup principle during the phase in which the excitation source is turned off. d) Standard device operation mode. Here the photovoltage buildup through time is demonstrated: “Turn on” is the point of time that the excitation source is turned on, and the “Turn off” time mark illustrates the point of time that the excitation source is turned off.

The working principle of the LS-NS device is briefly explained in Figure 1b and 1c (see Figure S3 for step-by-step charge carrier flow at different instances of the operation cycle). Once the device is excited, the photogenerated excitons in the CQW dissociate near the boundary of the aluminum. With the separation of electrons and holes at the boundary, holes are drawn into the aluminum side to a greater extent compared to electrons owing to the hole trapping sites, which are mostly located on the narrow facets.^15^ The net flow of holes to the aluminum layer results in a positive voltage build-up as presented in Figure 1d (Figure S3b), which is then followed by a resistance-capacitor (RC) decay due to the flow of the holes to ITO over the Shunt resistor (Figure S3c) until the charge becomes balanced, where we read zero voltage ultimately (Figure S3d). At this state, both aluminum and ITO (not grounded) are positively charged, where the CQWs are negatively charged due to the favored transport of holes compared to electrons to the aluminum. After the device reaches the zero-potential state under excitation, we observe a negative potential rise (Figure 1d) once we turn off the excitation source. Here, remaining electrons in the CQW layer will start to flow to the aluminum at the interface, charge the aluminum negatively, and lead to an increasing negative voltage as seen in Figure 1d. As time passes, the excess holes at the ITO will return back to the aluminum side and neutralize the whole device following RC decay (Figure S3e).

In this device architecture, orientation-controlled self-assembly of CQWs was implemented via a simplified Langmuir–Blodgett technique (see Supporting Information for details) to enhance the voltage build-up and sensitivity. For comparison, spin-coated films were optimized to match absorbance with that of the self-assembled single layer (∼20 nm). Nevertheless, spin coating produced nonuniform, web-like CQW clusters displaying largely random, predominantly face-down orientations (Figure S1c). Equal absorbance guarantees identical photon capture and exciton generation in both films; thus, any device-level response differences can be attributed mainly to the CQW orientation. In other words, the absorbance spectra of the edge-up and spin-coated films were matched during device preparation by controlling the thickness of the spin-coated film to generate the same number of excitons in the edge-up and face-down (random) oriented films for a given illumination. Therefore, any difference in sensitivity between the two can be attributed to what happens after absorption (i.e., charge separation and transport) rather than a difference in the number of excitons created.

As presented in Figure 2a, we prepared spin-coated and self-assembled CQW films with similar absorption intensities. The spectral sensitivities of both devices mostly follow behavior similar to the absorption spectrum of CQWs. However, toward shorter wavelengths, the rate of the increase in the sensitivity outpaces the increase of the absorption spectra. This indicates that the excess energy and momentum of hot charge carriers play a crucial role in facilitating the charge transfer at the CQW–Al interface, a phenomenon also benefiting the hot carrier solar cell architectures.^37^ If a standalone CQW particle or film is excited with a photon having higher energy than the bandgap, the excess energy excites the electron and hole to higher energy levels, and the hot carriers thermally relax to the band edges; however, at the metal–semiconductor interface, we utilize this excess energy and momentum in favor of the charge hopping, resulting in increased sensitivity.

**Figure 2:**
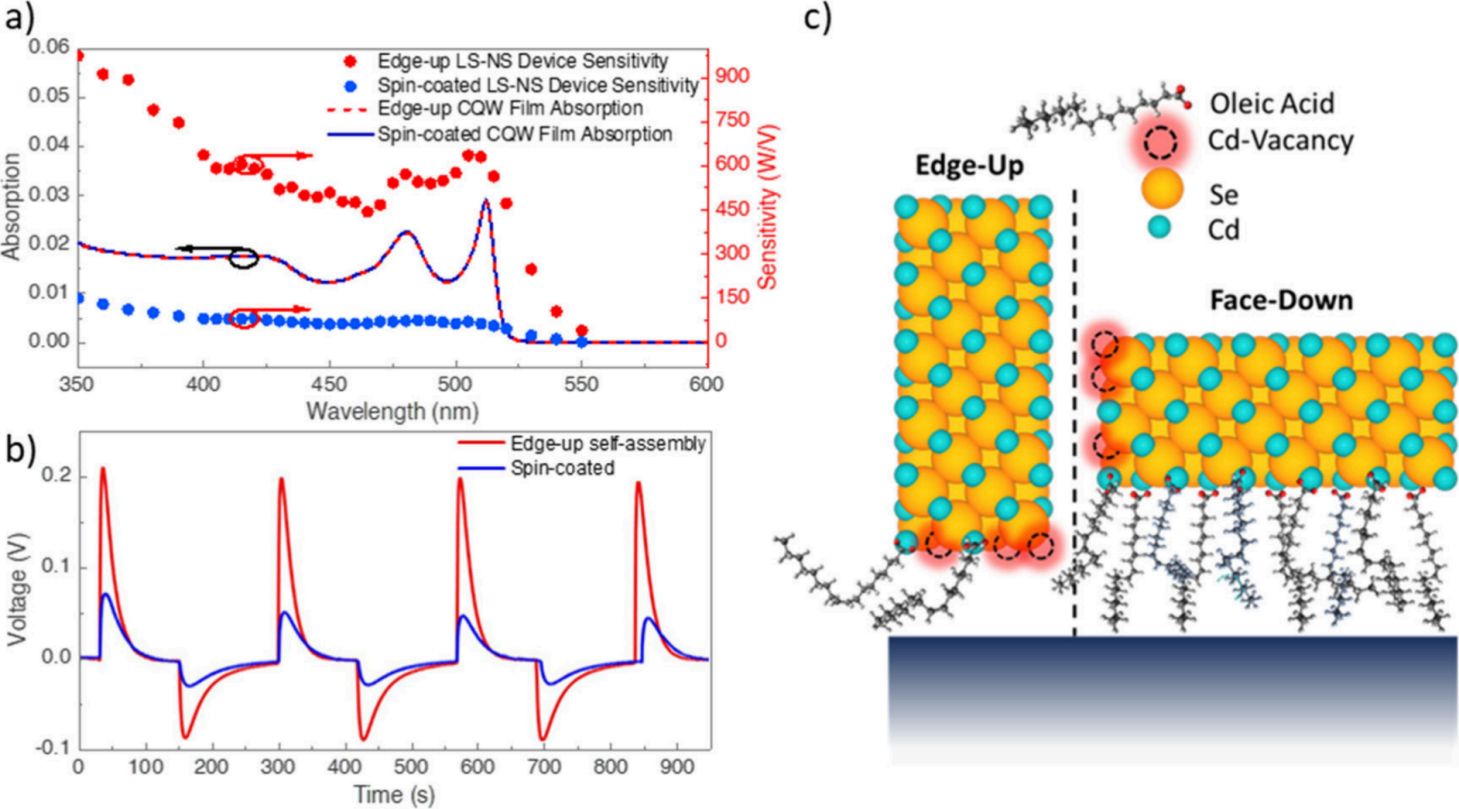
a) Spectral sensitivities of both spin-coated and self-assembled devices along with corresponding spectral absorption graphs. b) Consecutive standard operation of CQW LS-NS devices. c) Illustrative schematic of CQWs in contact with the Al. Crystal structure is underlined along with ligand distribution along the faces and edges with Cd vacancy sites.

In addition, despite the fact that each film has a similar number of CQWs, we observed that under the same excitation intensity of 0.86 W/cm^2^ the device with edge-up oriented film generates up to 4-fold more potential than the one with a spin-coated active layer as depicted in Figure 2b. This enhancement in potential build-up can be attributed to the localized trap sites on the periphery of CQWs. As depicted in Figure 2c, CQWs tend to have cadmium vacancies on the corners and edges, which exposes Se atoms^15,34,38–41^ known for accommodating hole trapping sites.^42^ Since these trap sites at the narrow facets are high in numbers,^38^ these trapped holes are localized closer to the metal contact in the case of edge-up orientation, which enhances the charge transfer at the interface. Such a trap-mediated hole transfer mechanism was also observed in quantum dot–organic hole acceptor systems.^43^ Additionally, these cadmium ligand vacancies on the periphery of the CQWs (narrow facets) result in lower ligand density and hence a weaker steric hindrance, which shortens the distance between the CQWs and aluminum layer, lowering the dielectric potential barrier. The relatively lower density of ligands on the periphery of CQWs was attributed to the low affinity of the ligands to the periphery because of the cadmium vacancies, as the L-type ligands used in the synthesis cannot bind to the exposed Se sites. In this case, there is more room for bending and rotation per ligand on the narrow facets owing to lower ligand density unlike the ligands on the large facets having high steric hindrance and brush-like straight arrangement. As a result, in the case of edge-up orientation, the freedom in conformation change of the ligands is reflected as a shorter distance between the CQW and metal layer. Therefore, in the case of edge-up orientation, carrier transfer at the contact interface is facilitated because of the hole trap sites and reduced CQW–metal distance as the charge transfer rate is exponentially dependent on the distance.^44^

In Figure 2b, we present the results of repetitive voltage–time measurements, conducted with an excitation at 405 nm and constant power of 0.86 mW. In this measurement, we started the excitation phase and waited until the device returned to the steady state under excitation. Once the potential drops to zero, we turned off the excitation source and waited until the negative voltage dissipated. Once the device was entirely neutral, we turned on the excitation source again, completing one cycle. We conducted four of those cycles consecutively to show the device’s repetitive operation and to compare the signal amplitudes and response times. We observed that the first cycle of each device has a slightly larger peak than the following three, and the following three peaks are at the same amplitude. This is because the long-living trap states do not return to their steady states before the next cycle starts and do not contribute to voltage buildup for the consecutive runs. Therefore, the first cycle has a slightly larger peak than the following ones. This process is shared between the edge-up and the spin-coated device. The LS-NS device with edge-up oriented CQW layer shows no significant change in photovoltage buildup as well as response time even after 1 h of uninterrupted on–off cycles, showcasing its stability for prolonged times (Figure S4).

Additionally, the spin-coated LS-NS device shows slower RC decays to the steady state than the edge-up devices even though it has a smaller signal amplitude. Furthermore, the edge-up device has a 4-fold larger signal amplitude than the spin-coated device during the optical excitation phase, where the holes are transferred to the metal. However, this enhancement of the signal is 3-fold when the excitation source is turned off, where the electrons are transferred from CQW to metal. This observation independently validates the above-mentioned reasoning that the cadmium vacancies are hole trap sites, which facilitate the charge transfer.

We have conducted excitation power-dependent voltage buildup measurements of those devices at different excitation powers at the same wavelength of 405 nm and compared their peak sensitivities for each voltage–time measurement. As presented in Figure 3a, the signal intensities of both devices increase with higher optical excitation powers. The voltage build-up reaches 600 mV with the LS-NS devices with an edge-up self-assembled CQW monolayer. This value of potential is three times higher than the best-reported value in the literature, which is obtained by sophisticated multilayer structured LS-NS devices exploiting exciton funneling.^30^

**Figure 3:**
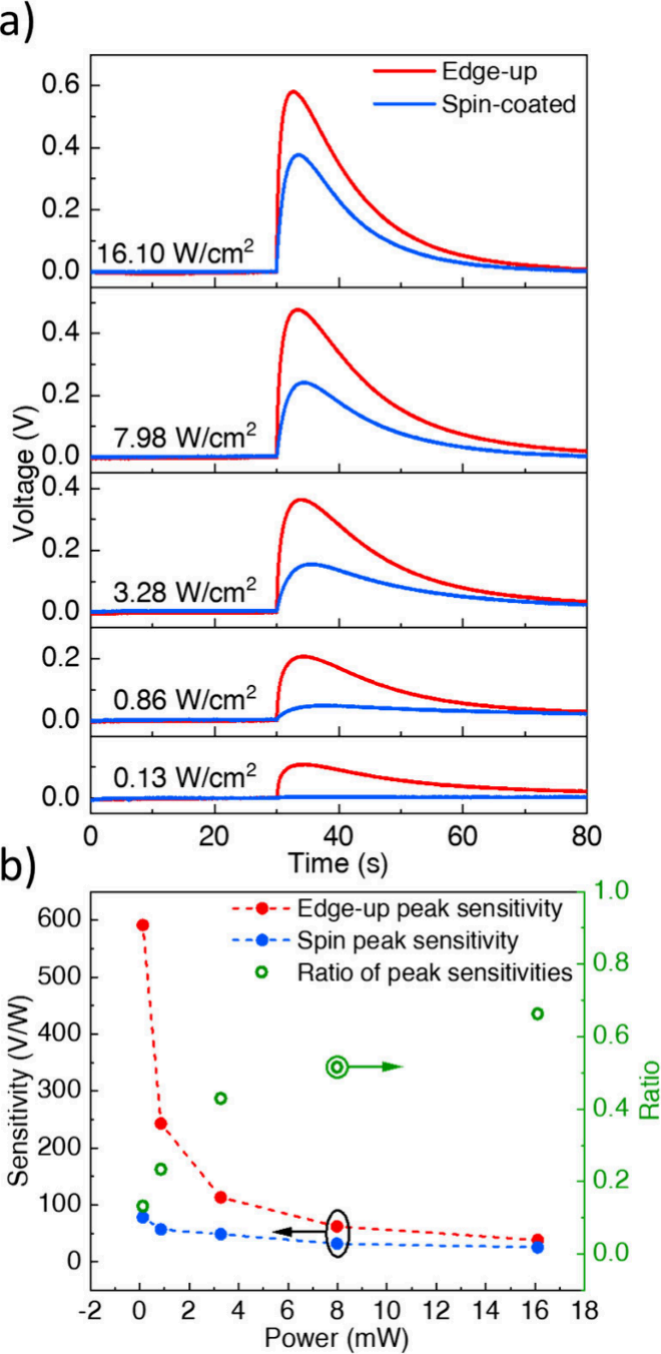
a) Photovoltage build-up curves of different excitation powers plotted on the same scale. b) Excitation power-dependent sensitivity graphs calculated through the peak signal of the corresponding photovoltage buildup shown along with the ratio of sensitivities (spin-coated/edge-up).

As presented in Figure 3b, the sensitivity of the devices follows an inverse relationship with the excitation intensity. Additionally, the ratio between signal amplitudes of the LS-NS devices with self-assembled CQW and spin-coated films decreases with the increasing excitation power, which is presented in Figure 3b as black rings. The drop of sensitivity as the excitation power increases can be attributed to the decrease in exciton dissociation rate because of the limited number of defect sites in our CQWs helping the charge trapping and hopping. As the excitation power increases, the density of electron–hole pairs generated in a given time also increases—eventually approaching or exceeding the density of trap sites. In that regime, traps can become saturated, and additional photogenerated carriers will recombine or equilibrate rather than contribute to the photovoltage. The filling of the defect sites available for charge dissociation under intense illumination^34^ causes a limitation for charge transfer to the metal layer, and hence this puts an upper limit on the voltage build-up. An optical analogous behavior was previously observed with 2.5 ML CQWs,^34^ where the CQWs with relatively low trap density show a saturation of trap emission with increasing excitation intensity, whereas the one with high trap density does not exhibit such saturation behavior.

Finally, we investigated the voltage decay behavior of LS-NS devices with self-assembled and spin-coated CQW layers. As presented in Figures 4a and 4b, we found that the RC decay times gradually decreased as the measurements were taken consecutively. Here, we turned off the excitation source as the voltage reached its maximum value. The first cycle of each device has a slightly larger peak than the following ones, which is because the long-living trap states could not return to their equilibrium before the next cycle starts and do not contribute to voltage build-up for the consecutive runs. This behavior is shared between the edge-up and spin-coated device. When we compare the decay times, the RC decay of devices with the edge-up self-assembled CQWs is more than three times faster than that of devices with the spin-coated CQWs, as can be seen in Figure 4. These faster dynamics, attributed to the edge-up orientation of CQWs, highlight a significant difference in the charge transfer across the metal–semiconductor interface. The observed decay behavior supports our earlier discussion, suggesting that the edge-up orientation enhances charge transfer by reducing the interlayer distance at the CQW–Al interface and gives access to the preferential placement of charge carriers.

**Figure 4:**
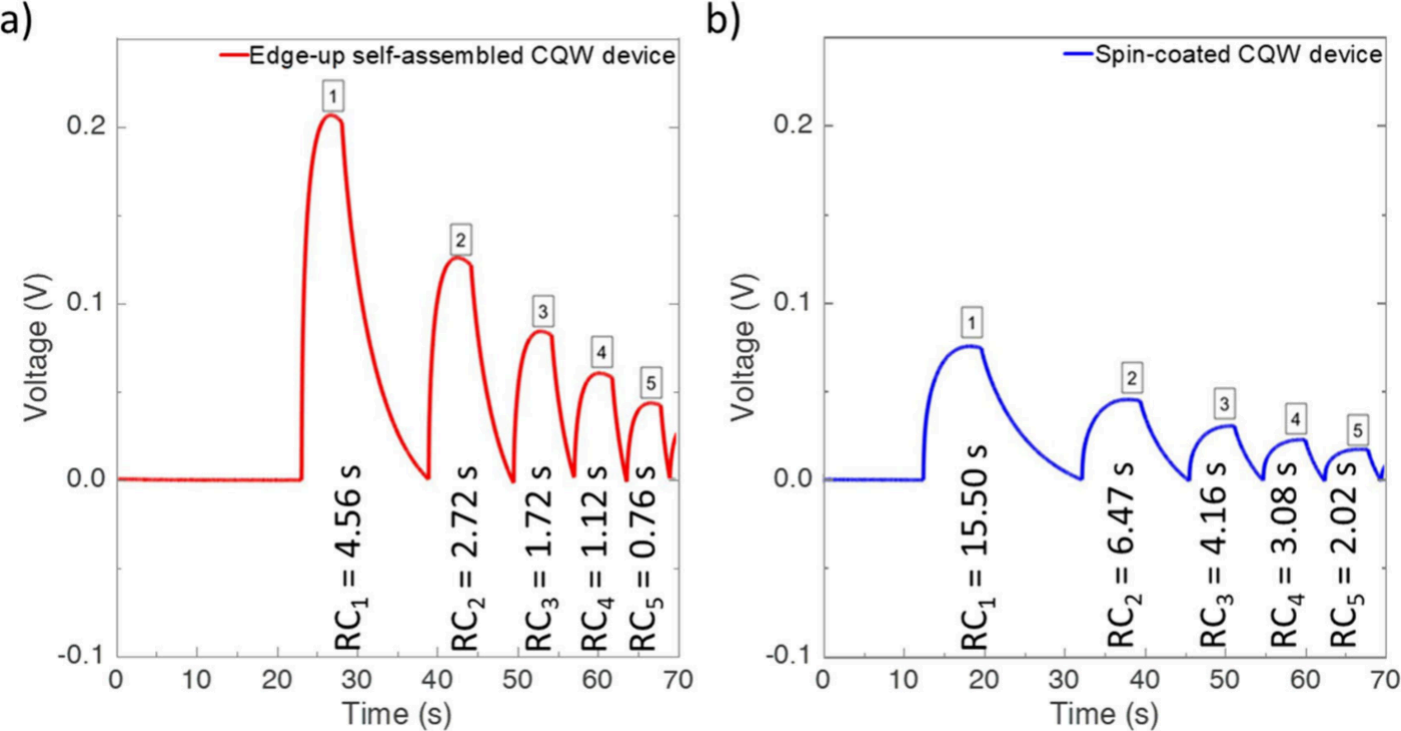
Repetitive decay curves of a) the edge-up oriented self-assembled CQW device and b) the spin-coated CQW device by the method of consecutive excitation turning of the source.

In conclusion, we developed LS-NS devices employing a single-layer edge-up oriented CQW film as the active absorber layer, which exhibits a record-high photovoltage buildup of 600 mV and a peak sensitivity of 600 V/W, approaching the performance of advanced architectures utilizing exciton funneling and plasmonic enhancements. In addition, we demonstrated the 8-fold improvement in the sensitivity compared to the LS-NS devices with a spin-coated colloidal semiconductor layer, showcasing the importance of the orientation of particles in device performance, which is a unique feature of CQWs. Such an enhancement is a result of the trap sites rich in numbers on the edges of the CQWs, which helps the hopping of the holes localized within close proximity to the metal–CQW interface. In addition, in the case of edge-up orientation, the reduced CQW–metal distance because of low ligand density further assists in the charge transfer rate and hence voltage build-up and three-times faster RC decay. Moreover, toward shorter wavelengths, we showed that the excess energy and momentum of hot charge carriers enhance the charge transfer at the CQW–Al interface. Our present work employs CdSe CQWs as a model system because they are well-studied and readily synthesized with high quality; however, the concepts demonstrated here are not limited to CdSe. In fact, the idea of orientation-controlled enhancement should be applicable to any colloidal semiconductor system with a spatial defect distribution anisotropy. Owing to the ease of fabrication allowing large area applications and working principles without requiring external bias, LS-NSs are promising device platforms for the implementation of light-sensing applications in large-area photosensitive windows and glass facades of smart buildings and smartphones. In addition to the orientation control, their performance can be further improved by implementing heterostructured semiconductor nanocrystals, especially the ones with type-II band alignment, which facilitate charge carrier separation and minimize their recombination.

## Supplementary Material



## Notes

Materials and methods; synthesis of 4.5 monolayer thick colloidal quantum wells, fabrication of light-sensitive nanocrystal skins, device characterization, self-assembly of colloidal quantum wells, spin-coating; band energy levels of the device layers; schematic for charge carrier flow in the device at different stages of working cycles of the LS-NS device; and stability test of the LS–NS device (PDF)
