# Supplementary material for: Highly Photosensitive Colloidal Quantum Well Based Nanocrystal Skins Assisted by Orientation Control
Source: Nano Lett. 2025 Nov 17;25(47):16601–7. doi: 10.1021/acs.nanolett.5c03234 (PMC12670507; doi:10.1021/acs.nanolett.5c03234)
Supplement: Supplementary file 1 [file nl5c03234_si_001.pdf]

Supporting Information

Highly Photosensitive Colloidal Quantum Well Based Nanocrystal Skins Assisted by Orientation Control

Furkan Isik,[1,4,5†] Taylan Bozkaya,[1†] Iklim Bozkaya,[1] Savas Delikanli,[1,2] Betul Canimkurbey[1,6] and Hilmi Volkan Demir[1,2,3*]

[1] UNAM − Institute of Materials Science and Nanotechnology and The National Nanotechnology Research Center, Bilkent University, Ankara 06800, Turkiye

[2] LUMINOUS! Center of Excellence for Semiconductor Lighting and Displays, School of Electrical and Electronic Engineering, School of Physical and Mathematical Sciences, School of Materials Science and Engineering, Nanyang Technological University, Singapore 639798, Singapore

[3] Department of Electrical and Electronics Engineering, Department of Physics, Bilkent University, Ankara 06800, Turkiye

[4] Institute of Accelerator Technologies, Ankara University, Ankara, 06830, Turkiye

[5] Turkish Accelerator and Radiation Laboratory (TARLA), 06830, Ankara, Turkiye

[6]Department of Physics, Polatlı Faculty of Arts and Sciences, Ankara Hacı Bayram Veli University, Ankara, 06900 Turkey

[†] These authors contributed equally.

* Correspondence to: hvdemir@ntu.edu.sg, volkan@bilkent.edu.tr

Synthesis of 4.5 monolayer thick colloidal quantum wells: The synthesis of colloidal quantum wells was carried out by following a previously reported recipe with slight modifications. 340 mg cadmium myristate, 24 mg selenium powder and 15 mL of octadecene were loaded into a three-neck flask along with a stirrer magnet. The mixture was vacuumed for 10 min at room temperature and then an additional 10 min at 90 °C. Then, the reaction atmosphere was set to nitrogen and the temperature was set to 240 °C. As the temperature hit 190 °C (when the color of the reaction mixture turned orange), 120 mg cadmium acetate dihydrate was added to the reaction. After 8 min of growth time at 240 °C, the reaction was quenched with the injection of 2 mL of oleic acid and placing a water bath under the flask. The purification of 4.5 monolayer thick colloidal quantum wells was achieved through selective precipitation by using hexane as solvent and ethanol as antisolvent.

Fabrication of light-sensitive nanocrystal skins: 100 nm thick ITO-coated glass substrates from Osilla with dimensions of 7.5 cm × 2.5 cm were cut into three even pieces. Then, we cleaned the substrates via sonication with Hellmanex solution, deionized water, acetone and isopropanol for 10 min each time. Following, we covered two opposite edges of each substrate piece with a Kapton tape (0.5 cm wide) to access the ITO contact when the tape was removed. Subsequently, we coated a 50 nm thick Al_2O_3 film at 300 °C using atomic layer deposition (ALD). After letting the samples cool to room temperature, we took them out and washed them with DI water, acetone, and isopropanol again. After this washing step, we repeated the same ALD growth to achieve 100 nm thick Al_2O_3 film. Then, we self-assembled the colloidal quantum wells by following the procedure explained in the main text. Finally, we coated the aluminum contact on the top with thermal evaporation. After the removal of Kapton tape, we ended up with an active area of 2.25 cm^2.

Device characterization: To measure voltage, we used a Keysight B1500A semiconductor parameter analyzer equipped probe station, positioning the samples on a glass stand and placing electrical contacts using probes at both the ITO and aluminum sides. A 405-nm LED, powered by an external power source, served as the excitation source. For spectral sensitivity measurements we incorporated a white LED with a custom-built monochromator. Optical excitation power was measured using an optical powermeter. The probes connected to the device were also linked parallel to a shunt resistor to regulate the discharge rate in a controlled manner.

Self-Assembly:

As illustrated in Figure S1a, we placed our alumina coated substrate in a Teflon dish with a diameter of 6 cm and a depth of 1.5 cm, and filled it with a polar solvent (ethylene glycol, EG) as the subphase until the air-liquid interface is 2 mm above the substrate. Then, we dropped 10 µL of CQW-octane dispersion with a concentration of 24 mg/mL on the inner wall of the Teflon dish so that the CQW dispersion spreads over the interface uniformly. Directly dropping the CQW dispersion on the liquid subphase results in formation of ripples, which impairs uniform film formation. Following, we closed the Teflon dish with a lid to slow down the evaporation of octane and provide enough time for the CQWs to reach their thermodynamically favored configuration. Edge-up configuration is obtained through the interplay between the polarity of

subphase, ligands of CQWs, evaporation rate and concentration of CQWs. The orthogonality of polarity between the subphase and the exposed part of the ligands is crucial to achieve edge-up orientation, where the higher difference in polarity results in weaker attractive interaction between subphase and CQWs, and energy is minimized through maximization of CQW-CQW van der Waals interactions. This results in large facets of CQWs facing each other and smaller facets contacts with the subphase if enough time is provided through slow evaporation of the solvent. As reported in our previous work, using diethylene glycol, a less polar subphase, and hexane, a more volatile solvent, results in face-down oriented self-assembly of same CQWs[8,23]. Once the solvent is fully evaporated, the subphase was drained with a syringe, through a hole on the bottom side of the Teflon dish, to transfer the CQW film on the substrate. As the last step of the self-assembly process, we placed the sample in a desiccator connected to vacuum to remove remaining subphase. The scanning electron microscopy (SEM) image resulting edge-up oriented closed pack CQW film is presented in the Figure S1b.

Spin Coating:

For the spin-coated samples, we first measure the spectral absorbance of the edge-up coated film in order to adjust the spin coating parameters, specifically amount of CQW dispersion, spin speed, spin duration and solvent, for ensuring similar CQW amount on both films. The best results, in terms of surface coverage and uniformity, were achieved using 50 µL of CQW dispersion (having a concentration of 24 mg/mL) with hexane as solvent and spinning at 2,500 rpm for 30 s. Despite all the effort, the spin-coated films ended up as a “web-like” structure with clusters of CQWs which can be seen in the SEM image presented in Figure S1c. This cluster formation leads to a non-uniform film that lacks surface coverage. At the same time, when we look closer at those clusters, we realize that CQWs are randomly oriented between the orientations of edge up and face down; however, one can say the amount of the edge up oriented CQWs in these assemblies is respectively low.

Since we are working at a regime of single-layer films for the case of self-assembly, which has a thickness near 20 nm depending on the dimensions of the CQWs, it is difficult to achieve the same thickness with spin-coating all around the film considering the nature of this thin film method with nanoparticles. Hence, we chose the absorption of these films as a reliable empirical reference point to compare the device performances. The exact level of absorption on two films indicates the same number of photons are absorbed. Given that both films are composed of the same material, and the primary difference is the orientation of the CQWs, a comparable number of excitons should be generated in each. Hence, any variations in the signal between the two devices can more precisely reflect differences in the charge dissociation and transfer efficiency at the metal-semiconductor interface.

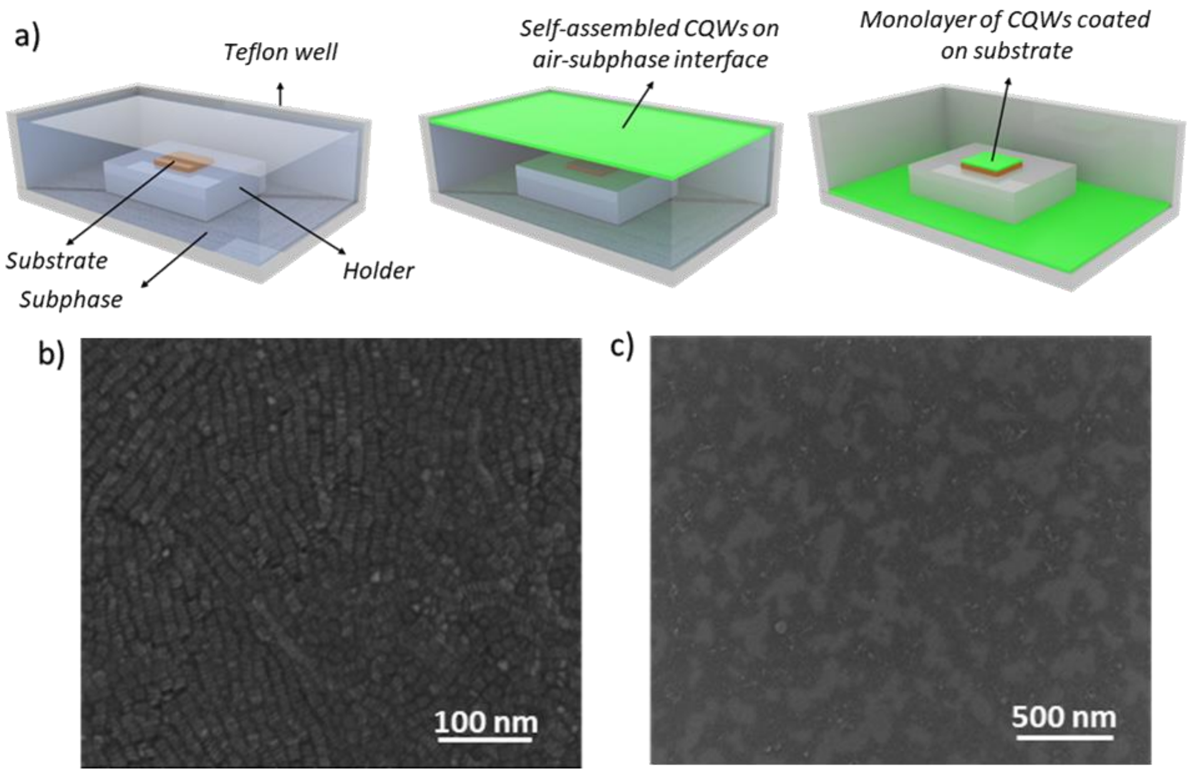

Figure S1 a) Process flow schematic of the self-assembly process. b) Scanning electron microscope (SEM) image of monolayer edge up self-assembled 4ML CdSe CQW film. c) Scanning electron microscope image of absorption matched (to edge up film) spin coated film of 4ML CdSe CQWs.

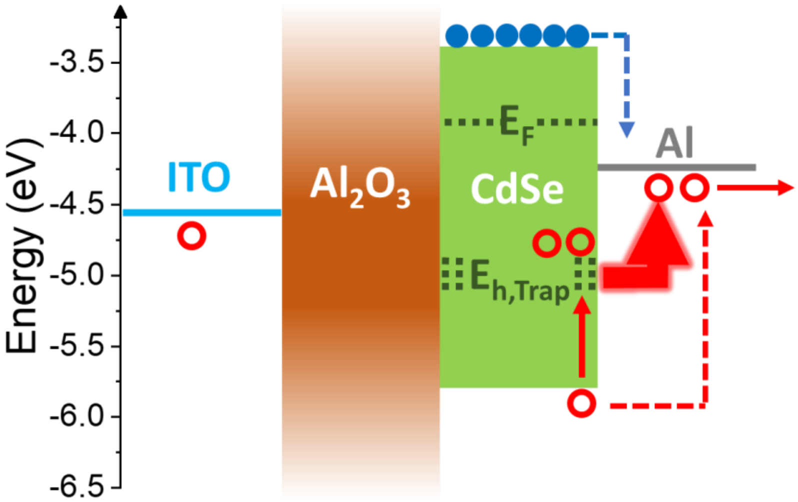

Figure S2 Band energy levels of the layers and charge carrier flow in the device at the instance of photoexcitation.

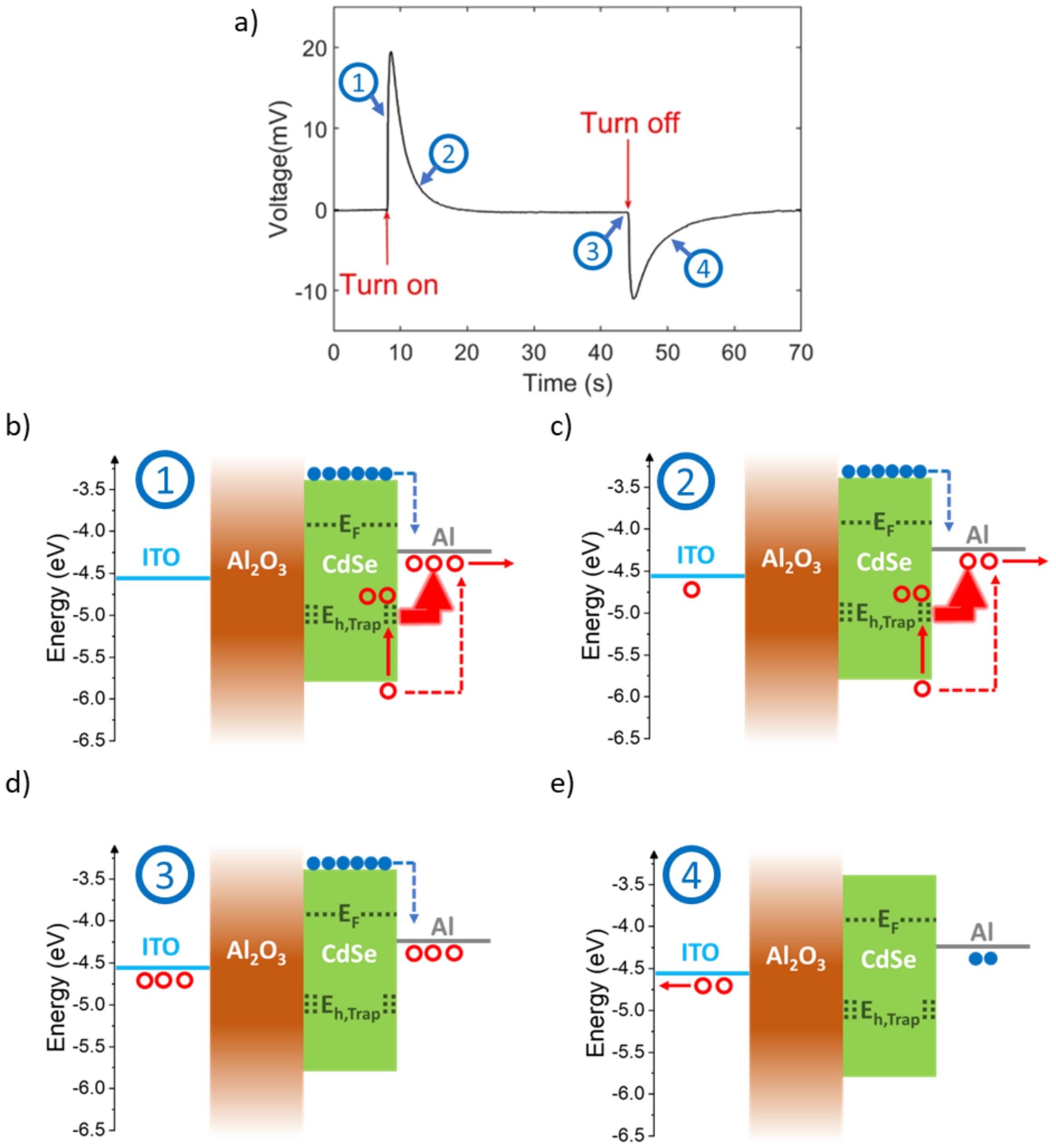

Figure S3: Charge carrier flow in the device at different stages of working cycle of LS-NS device.

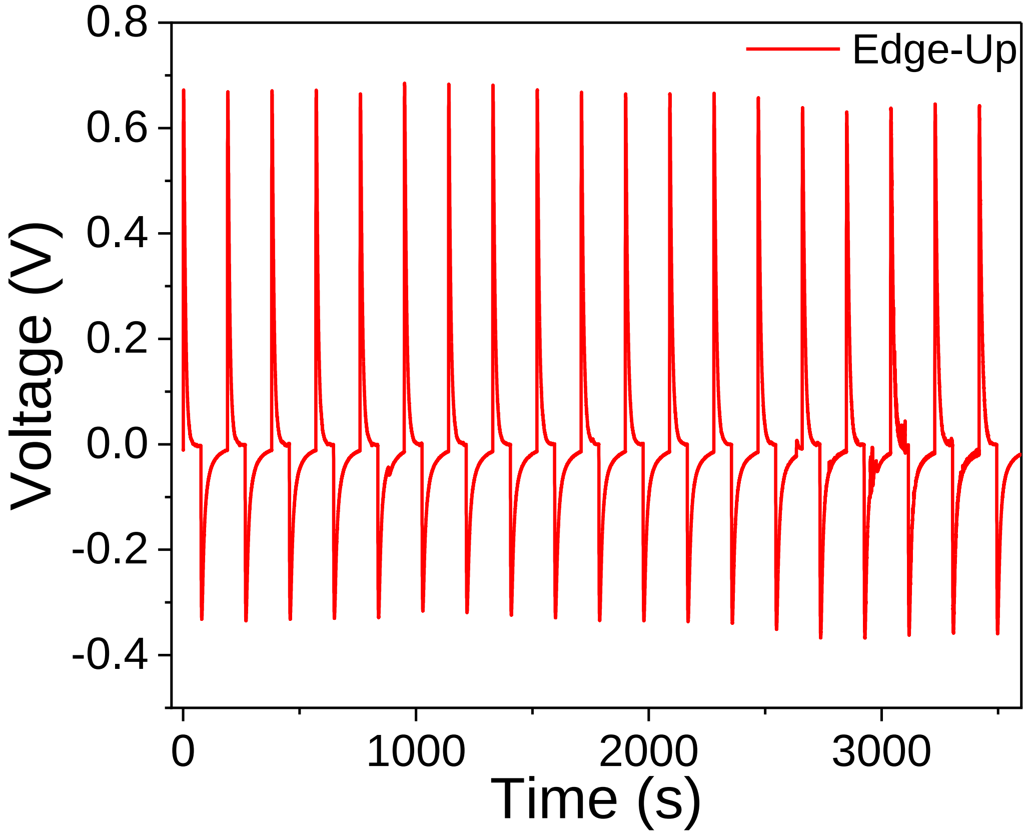

Figure R7: Consecutive standard operation of LS-NS device with edge-up oriented self-assembled CQW layer over 1 h of operation time.
